# Effect of MK-801, an antagonist of NMDA receptor in the pedunculopontine tegmental nucleus, on cardiovascular parameters in normotensive and hydralazine hypotensive rats

**DOI:** 10.22038/IJBMS.2022.62431.13809

**Published:** 2022-05

**Authors:** Mohammad Reza Hosseiniravesh, Vida Hojati, Abolfazl Khajavirad, Hooman Shajiee, Mohammad Naser Shafei, Reza Mohebbati

**Affiliations:** 1 Department of Biology, Damghan Branch, Islamic Azad University, Damghan, Iran; 2 Department of Physiology, Faculty of Medicine, Mashhad University of Medical Sciences, Mashhad, Iran; 3 Applied Biomedical Research Center, Mashhad University of Medical Sciences, Mashhad, Iran; 4 Division of Neurocognitive Sciences, Psychiatry and Behavioral Sciences Research Center, Mashhad University of Medical Sciences, Mashhad, Iran

**Keywords:** Blood pressure, Glutamate, Hypotension, NMDA receptor, Pedunculopontine - tegmental nucleus

## Abstract

**Objective(s):**

In the present study, the cardiovascular effects of glutamate NMDA receptor of the pedunculopontine tegmental nucleus (PPT) in normotensive and hydralazine (HLZ) hypotensive rats were evaluated.

**Materials and Methods:**

In the normotensive condition, MK-801(1 nmol; an NMDA receptor antagonist) and L-glutamate (L-Glu, 50 nmol an agonist) alone and together were microinjected into the nucleus using a stereotaxic device. In hypotensive condition, 2 min after induction of hypotension by HLZ (10 mg/kg, intravenous), drugs, same as in normotensive condition, were microinjected into the PPT. Recorded mean arterial pressure (MAP), systolic blood pressure (SBP), and heart rate (HR) were recorded throughout the experiment by a Power lab apparatus that was connected to a catheter inserted into the femoral arty. The cardiovascular changes (Δ) induced by microinjection drugs were computed and statistically analyzed.

**Results:**

In the normotensive condition, L-Glu significantly increased ΔMAP and ΔSBP (*P*<0.001) and decreased ΔHR (*P*<0.01) compared with the control. MK-801 alone significantly increased HR (*P*<0.05) while co-injected with L-Glu + MK-801 it significantly attenuated the L-Glu effect on ΔMAP and ΔSBP but augmented ΔHR (*P*<0.01). In the hydralazine hypotension condition, L-Glu significantly improved hypotension (*P*<0.01) and deteriorated bradycardia induced by HLZ (*P*<0.05). MK-801 alone did not significantly affect ΔMAP, ΔSBP, and ΔHR but when co-injected with L-Glu (L-Glu + MK-801) it could significantly attenuate the cardiovascular effect of L-Glu in the PPT.

**Conclusion:**

We found that activation of NMDA receptors of the glutamatergic system in the PPT evoked blood pressure and inhibited HR in both normotensive and hypotensive conditions in rats.

## Introduction

The pedunculopontine tegmental nucleus (PPT) is an irregularly shaped nucleus located at the upper brainstem which was first introduced as part of the reticular activating system of the brainstem. The structure of the PPT is heterogeneous, it has several neurons and has many projections to different areas of the brain from the forebrain to the spinal cord (1). This area consists of two parts: Dense pars or pars compacta (PPTc), which contains a high density of cholinergic neurons, and diffuse part (PPTd) or Pars dissipate, which contains glutamatergic and cholinergic neurons (2-4). Based on the electrophysiological studies, three cell types have been identified in the PPT nucleus (4). The first type is characterized by continuous and fast-acting phase potentials that can be activated by the depolarization current and are probably glutamatergic (5, 6). The second type of neurons creates single action potentials with larger subsequent hyperpolarization in response to the depolarization current. About 50% of type 2 neurons are cholinergic and the third neurons also have characteristics of the two previous neurons (6, 7). There is a large number of neurotransmitters in this nucleus that control the functions of the nucleus. The most important of these neurotransmitters are glutamate, GABA, and acetylcholine (4, 7). Glutamate, especially its NMDA receptor, was identified by physiological studies in the PPT, and it has shown that injection of glutamate into this nucleus activates neuronal firing related to movement. It also documented that injection of carbachol (an acetylcholine agonist) in a dose-dependent manner causes hyperpolarization and decreased cholinergic neuronal activity, and this effect is blocked by atropine (8). Injecting bicuculline, a GABA antagonist, also increases the motility and inhibition of cholinergic neurons (9). Currently, most studies are focused on the cholinergic effects of this nucleus on movement and sleep (10, 11). 

There are few studies on the role of the PPT in cardiovascular activity. Padley *et al*. have shown that stimulation of the PPT nucleus increases cardiovascular activity and this effect is mostly mediated by the PPT projection to the rostral ventrolateral medulla (RVLM), an important integrative area for cardiovascular regulation (12, 13). Topchiy *et al*. also reported that glutamate microinjection into the PPT increased blood pressure(BP), heart rate( HR), and respiratory rate (14). We previously also evaluated the effect of GABAergic, nicotinic receptors of cholinergic and nitrergic systems of this nucleus (15-17) on the cardiovascular system. 

The glutamatergic system is an excitatory system that presents in numerous brain areas (18). The important functions of this system in the brain are modulation of pain, memory, movement, sleep, and cardiovascular regulation in normotensive and hypotension induced by hemorrhage (19). The inotropic receptors of this system are well-known receptors in the brain which are classified into two N-Methyl-D-aspartate (NMDA) and non-NMDA receptors (18). It has been documented that L-glutamate (L-Glu) via its inotropic receptors plays an important role in the regulation of the cardiovascular system (18). The cardiovascular effect of L-Glu in several brain areas such as the paraventricular nucleus (PVN), periaqueductal gray (PAG), and A5 area has been shown (20-22). Previous studies also showed that injection of L-Glu into the cuneiform nucleus (CnF) evoked two long and short pressor responses and the role of the NMDA receptor was critical (23). A primary study also reported that injection of L-Glu into the PPT elicits various functions such as apnea, tachypnea, hypertension (HTN), and sinus tachycardia (14). The role of the glutamate system in the regulation of cardiovascular function during hypotension also has been reported. For example, in a recent study, we showed the involvement of the vlPAG glutamatergic system in the regulation of cardiovascular parameters in normotension and hypotension induced by hemorrhage(24). Although the glutamatergic system is present in the PPT its effect on cardiovascular function is not determined, therefore, the current study is designed to determine the probable cardiovascular effect of NMDA receptor of the glutamatergic system of the PPT in normotensive and hypotension induced by hydralazine (HLZ). 

## Materials and Methods

***Animals***

In this study, 40 male Wistar rats (250 ± 20 g) were used. The animals were provided from the animal house of the faculty of medicine of Mashhad University of Medical Sciences (MUMS) and maintained in a standard environment and had access to food and water. Our experimental procedures were done in accordance with the guidelines of the ethical committee of Mashhad University of Medical Sciences (IR.MUMS.MEDICAL.REC.1401.020).

***Arterial cannulation and blood pressure recording***

 In this experiment, rats were anesthetized with urethane (1.5 g/kg, IP)(25). After confirmation of anesthesia, the femoral artery was cannulated for recording of the cardiovascular parameters. For this purpose, an incision was done in the femoral region in the internal abdomen to expose the femoral nerve, artery, and vein. The artery was isolated and a blue angiocatheter filled with heparinized saline (50 u/ml) was inserted and advanced about 3 cm into the artery, then with 4-0 silk suture fixed (24). The angiocatheter by a blood pressure transducer was connected to a Power Lab instrument (Australia), and cardiovascular parameters including mean arterial pressure (MAP), systolic blood pressure (SBP), and heart rate (HR) were recorded (26).

***Drug microinjection***

For injection of drugs into the nucleus, rats were mounted on a stereotaxic instrument (Stoelting, USA). Then the scalp was cut and Bregma and Lambda were exposed, according to the coordinations of Paxinos and Watson atlas (AP: 7.44-8.64, L: 1.6-2.2, H: 6.8-7.8), a small hole (2 mm in diameter) was made just above the PPT nucleus (27). For injection of drugs, we used a single-barreled micropipette with 40–45 μm in tip diameter that, via a polyethylene tube, was connected to a manual microinjector (Harvard). The micropipette was carefully inserted into the PPT, and after that by screwing the microinjector, the desired volume (100–150 nl) of the drug was injected into the nucleus within 30 sec (25).

***Drugs***

Urethane (merk Germany), L-glutamate (L-Glu, an agonist, Sigma, USA), MK-801 (an NMDA antagonist, Sigma, USA), and hydralazine (HLZ, Sigma, USA) were used in this study. All drugs were dissolved in saline.

***Animal groups***

In this study animals were randomly divided into 8 groups (n= 6 for each group) as follows:

1) Control group: Saline microinjected into the PPT

2) L-Glu group: The L-Glu (50 nmol) (28) microinjected into the PPT

3) MK-801 group: MK-801(1 nmol) (29) was microinjected into the PPT

4) MK-801+ L-Glu: MK-801 and, 2 min later, L-Glu microinjected into the PPT

5) Hydralazine: HLZ (10 mg/kg (30) intravenously injected

6) HLZ+ L-Glu: 2 min after induction of hypotension by HLZ (10 mg/kg, IV), L-Glu microinjected into the PPT

7) HLZ+ MK-801: 2 min after induction of hypotension by HLZ, MK-801 microinjected into the PPT

8) HLZ+ L-Glu + MK-801: 2 min after induction of hypotension by HLZ, MK-801 and L-Glu microinjected into the PPT

 Injection volume in all groups was 100–150 nl (16)

***Data analysis***

The data were calculated and expressed as mean ± SEM. After recording SBP, MAP, and HR, those changes (∆) (difference between before and after injection of drugs) were computed and analyzed. For comparison changes of parameters several times, repeated measure ANOVA and for maximal changes, One-way ANOVA with Tukey’s *post-hoc* test were used. *P*<0.05 was used to indicate statistical significance.

## Results

***Cardiovascular values induced by microinjection of saline into the PPT in normotensive anesthetized rats***

To determine the effect of saline on cardiovascular values, the changes induced by microinjection of saline into the PPT were evaluated. Before microinjection of saline, MAP, SBP, and HR were 104.5 ± 1.87 mmHg, 110.23 ± 3.3 mmHg, and 324.98 ±7.43 beats/min, respectively. However, microinjection of saline did not change those parameters compared with before injection (MAP: 99.60 ± 4.4 mmHg, SBP: 105.65± 3.97 mmHg, and HR: 320.98 ± 8.45 beats/min).

***Cardiovascular values induced by microinjection of L-glutamate and MK-801 into the PPT in normotensive rats***

**Figure 1:**
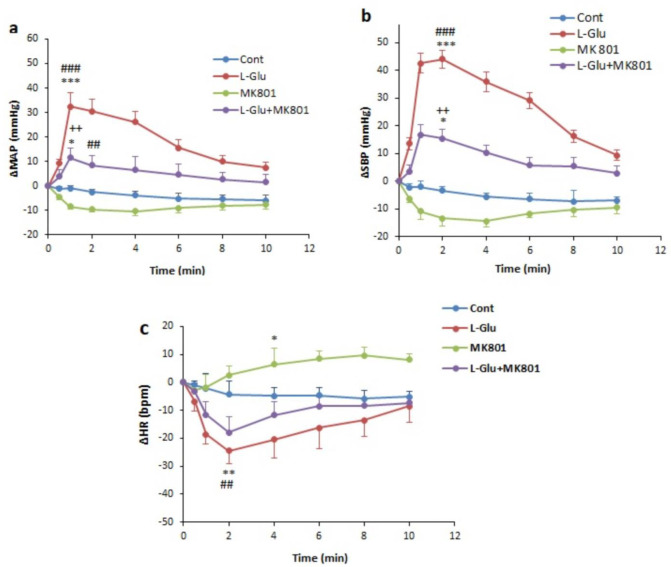
Time course changes of mean arterial pressure (∆MAP) (a), systolic blood pressure (∆SBP) (b), and heart rate (∆HR) (c), after microinjection of L-Glu (50 nmol), MK-801(1 nmol), and L-Glu + MK801 into the PPT in normotensive rats. Repeated measures ANOVA, with Tukey's *post hoc* test

**Figure 2:**
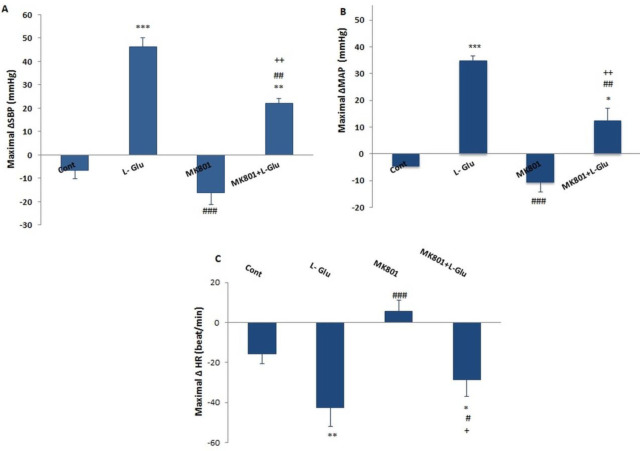
Maximal changes of ∆SBP (A), ∆MAP (B), and ∆HR (C), after microinjection of saline, L-Glu (50 nmol), MK-801(1 nmol), and co-injection of L-Glu + MK801 into the PPT nucleus in normal conditions. The data were expressed as mean ± SEM. ∆MAP: Mean arterial pressure, ∆SBP: Systolic blood pressure, ∆HR: Heart rate. Differences with *P*-value<0.05 were considered significant

In this experiment, L-Glu was microinjected into the PPT and changes in values were obtained. The time-course changes depicted that L-Glu significantly increased ∆SBP and ∆MAP compared with the control over time (*P*<0.001, Figure 1) while ∆HR significantly decreased compared with the control group over time (*P*<0.01). Maximal ∆SBP and ∆MAP also significantly increased (*P*<0.001) and maximal changes of ∆HR significantly decreased (*P*<0.01) compared with the control group (Figure 2). 

 Microinjection of MK-801 into the PPT nucleus did not significantly reduce ∆MAP and ∆SBP and enhanced ∆HR (Figure 1). To ensure the cardiovascular effect of the NMDA receptor in the PPT, in a separate group, firstly MK-801 was microinjected and 2 min later, L-Glu (L-Glu+ MK-801 group) was microinjected into the PPT nucleus. Results show that ∆MAP and ∆SBP in the presence of MK-801 significantly reduced compared with L-Glu alone over time (*P*<0.01, Figure 1). ∆HR also in the L-Glu+MK801 group was higher than in the L-Glu group but this effect was not significant. 

Maximal changes of ∆SBP and ∆MAP after pretreatment with MK-801 were significantly lower than those of the L-Glu alone group (*P*<0. 01, Figures 2a and b), but changes in ∆HR were non significantly higher compared with the L-Glu group (Figure 2c). Changes in ∆MAP, ∆SBP, and ∆HR in the L-Glu+MK801 group also were more significant than in the control group (*P*<0.5 to *P*<0.01). 

***Cardiovascular values induced by intravenous injection of hydralazine***

**Figure 3:**
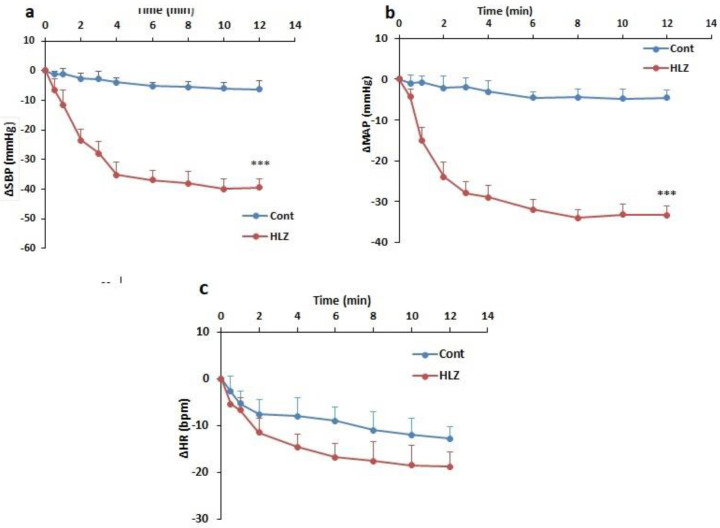
Time course changes of the mean arterial pressure (∆MAP) (a), systolic blood pressure (∆SBP) (b), and heart rate (∆HR) (c), after injection of saline and hydralazine (HLZ,10 mg/kg, iv), compared with the saline group. Repeated-measures ANOVA, with Tukey's* post hoc* test

To induce hypotension, HLZ was intravenously injected and changes in cardiovascular parameters were evaluated. As has been shown in Figure 3, ∆MAP, ∆SBP, and ∆HR in the HLZ group several times were lower compared with the control group over time (*P*<0.001) but only ∆MAP and ∆SBP were significant and changes in ∆HR were not significant (Figure 3).

**Figure 4:**
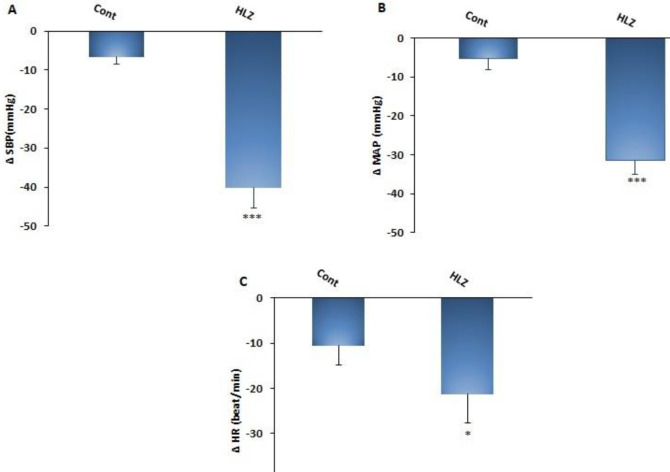
Maximal changes of ∆SBP (A), ∆MAP (B), and ∆HR (C) after injection of saline and hydralazine (HLZ,10 mg/kg, IV), compared with the saline group. One-way ANOVA, with Tukey's *post hoc* test

The maximal changes of ∆MAP, ∆SBP (*P*<0.001), and ∆HR (*P*<0.05) in the HLZ group were also significantly lower compared with the control group (Figure 4).

***Cardiovascular effect induced by L-glutamate, MK-801, and MK-801 +L-glutamate microinjected into the PPT in hydralazine hypotensive rats***

**Figure 5.**
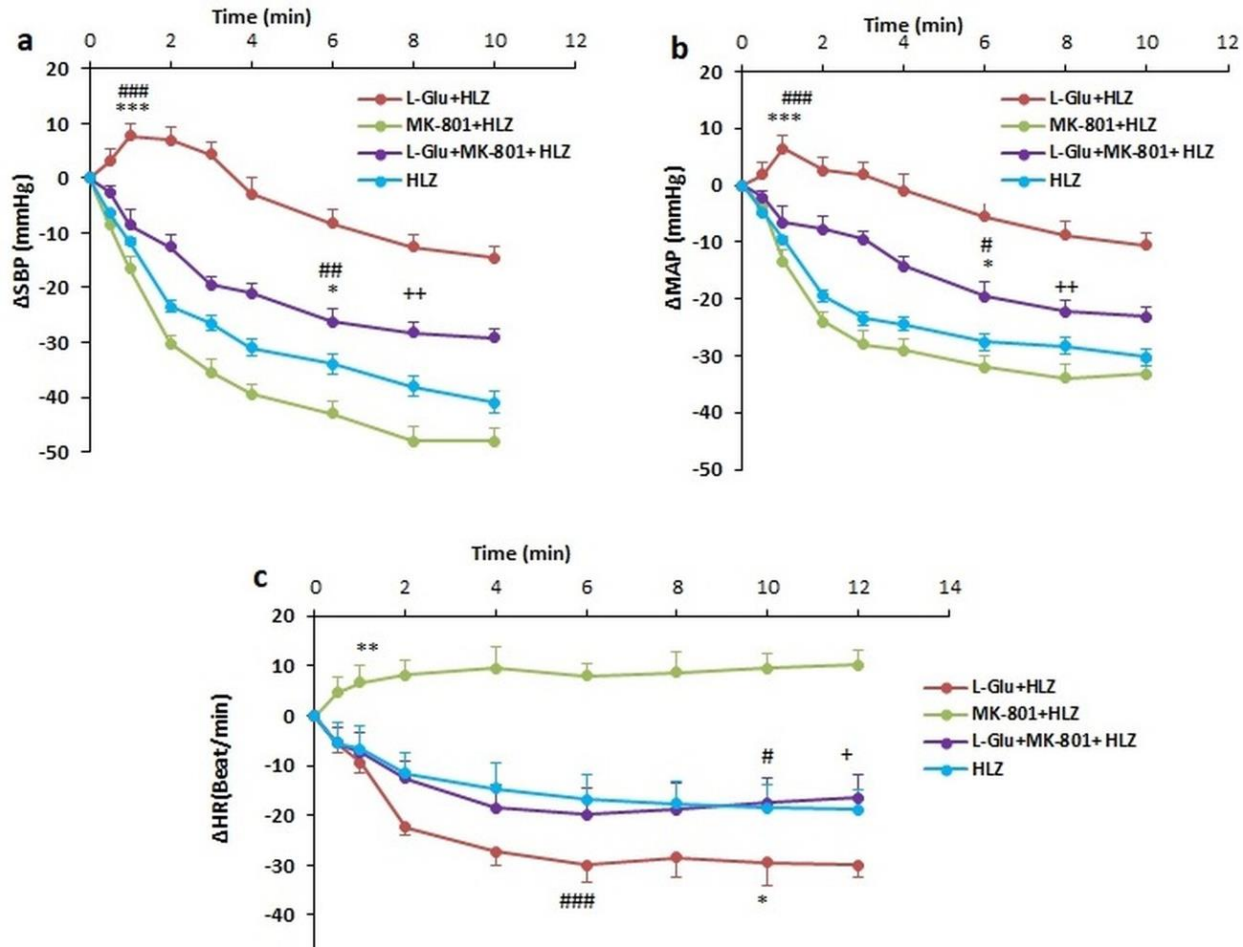
Time course changes of mean arterial pressure (∆MAP) (a), systolic blood pressure (∆SBP) (b), and heart rate (∆HR) (c) after microinjection of L-Glu (50 nmol), MK-801(1 nmol), and L-Glu + MK801 into the PPT in hydralazine hypotensive rats. Repeated-measures ANOVA, with Tukey's *post hoc test*

In this experiment firstly hypotension was induced by injection of HLZ (10 mg/kg, IV) and then drugs were microinjected into the PPT. Time course of results indicated that microinjection of MK-801 reduced ∆MAP and ∆SBP and increased ∆HR compared with HLZ but only changes in ∆HR were more significant than in the HLZ group (*P*<0.01, Figure 5). Injection of L-Glu in presence of HLZ (L-Glu+HLZ group) significantly decreased ∆HR and increased ∆MAP and ∆SBP with respect to HLZ and MK-801+HLZ groups over time (*P*<0.05 to *P*<0.001 Figure 5).

 In the L-Glu_MK-801+ HLZ group, ∆SBP and ∆MAP were significantly higher than in the HLZ group (*P*<0.05) and significantly lower than in the L-Glu+HLZ group (*P*<0.01). Changes in ∆HR in this group also were not significant as in the HLZ group while it was increased compared with L-Glu+ HLZ (*P*<0.05, Figure 5). Changes in ∆HR in the L-Glu + MK-801 + HLZ group were also significantly lower than in the MK-801 + HLZ group (*P*<0.01). 

During HLZ induced hypotension, maximal changes of ∆SBP and ∆MAP after microinjection MK-801 (MK-801+ HLZ group) appeared, not significantly lower than HLZ group but maximal changes of ∆HR significantly increased (*P*<0.01) compared with the HLZ group. 

**Figure 6:**
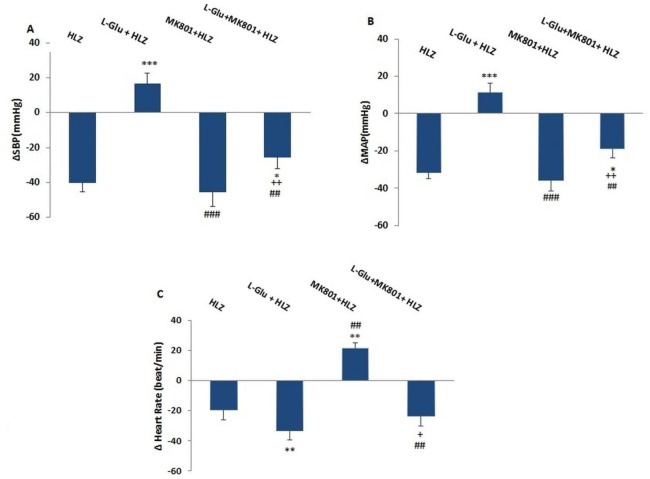
Maximal changes of ∆SBP (A), ∆MAP (B), and ∆HR (C), after microinjection of L-Glu (50 nmol), MK-801(1 nmol), and co-injection of L-Glu + MK801 into the PPT nucleus in hydralazine hypotensive rats. The data were expressed as mean ± SEM. ∆MAP: Mean arterial pressure, ∆SBP: Systolic blood pressure, and ∆HR: Heart rate

In L-Glu+HLZ group maximal changes of ∆SBP and ∆MAP significantly amended compared with HLZ and MK-801+HLZ groups (*P*<0.001) while ΔHR significantly decrease than HLZ group. Co-injection of L-Glu and MK-801 in presence of HLZ (L-Glu + MK-801+HLZ) significantly decreased ∆SBP and ∆MAP induced by HLZ (*P*<0.05), but did not significantly augment bradycardia induced by HLZ (*P*>0.05). ∆SBP and ∆MAP in this group were also significantly lower than L-Glu (*P*<0.01, Figures 6-A and B). ∆SBP and ∆MAP in L-Glu + MK-801+HLZ group was also significantly higher than in MK-801+HLZ group (*P*<0.05, Figure 6).

*P<0.05 ,**P<0.01 and *** *P*<0.001: compared with control. ## *P*<0.01 ### *P*<0.001: compared with MK-801

++: *P*<0.01: compared with L-Glu

***: *P*<0.001, **: *P*<0.01, and *: *P*<0.05 vs the control group. ###: *P*<0.001, ##: *P*<0.01 vs the L-Glu group, and ++: *P*<0.01 and +: *P*<0.05 vs the MK-801 group

*** *P*<0.001: compared with control

***: *P*<0.001 and *: *P*<0.05 vs the control group

**P<0.01 and *** *P*<0.001: compared with hydralazine. ###: *P*<0.001, ##: *P*<0.01, and #*P*<0.05: compared with MK-801+HLZ

++: *P*<0.01: compared with L-Glu+HLZ

***: *P*<0.001, **: *P*<0.01, and *: *P*<0.05 vs the HLZ group. ###: *P*<0.001 and ##: *P*<0.01 vs the L-Glu +HLZ group, and ++: *P*<0.01 and +: *P*<0.05 vs the MK-801 +HLZ group

**Figure 7:**
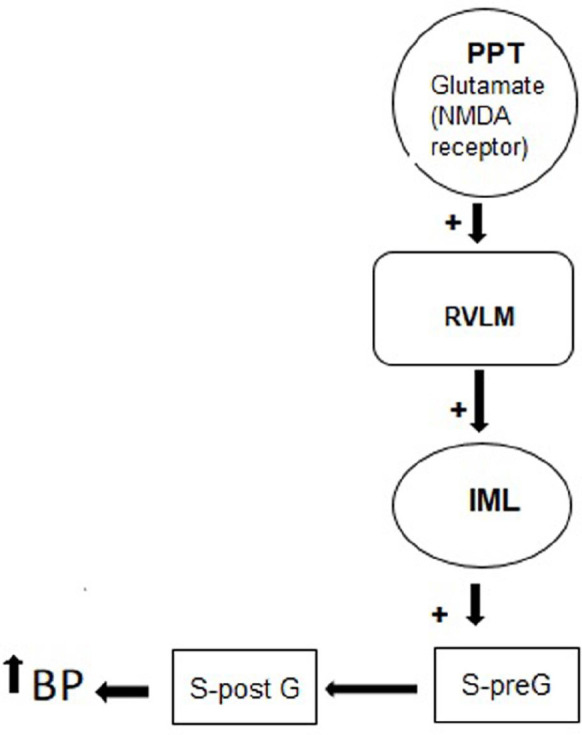
A postulated neural system for the cardiovascular effect of NMDA receptor of the PPT

RVLM: main sympato-excitatory area for cardiovascular regulation that receives input from several areas including PPT.

PPT: pedunculopontine tegmental nucleus; IML: intermediolateral cell column of spinal cord

S Pre G: sympathetic preganglionic neurons; S Post G: postganglionic sympathetic neurons

BP: blood pressure

## Discussion

In this research, the effect of the glutamatergic system of the PPT nucleus on the cardiovascular system in two normotensive and hypotensive rats was evaluated. We observed that in both conditions, when L-Glu was microinjected into the PPT nucleus, it caused an increase in blood pressure and decrease in HR but microinjection of the MK-801 alone into this nucleus did not significantly decrease MAP and SBP and increased HR in the control group. In addition, microinjection of MK-801 before L-Glu could significantly attenuate cardiovascular responses induced by L-Glu. Also, in hypotension conditions, the L-Glu similar to normotensive rats had an excitatory effect on blood pressure and an inhibitory effect on HR.

There are several pieces of evidence that the glutamatergic system is involved in central cardiovascular regulation in both normotensive and hypotensive conditions (23, 24, 31). Because the presence of the glutamatergic system in the PPT has been reported (8), we suggest that this system of the PPT has a cardiovascular function. Our results also confirm this role of the glutamatergic system of the PPT. This result is similar to a previous study that shows microinjection of glutamate into the PPT caused apneic responses, hypertension, and electroencephalographic responses (14).

The PPT is a heterogeneous nucleus divided into sub-regions and each one precipitates in a specific function. It has been documented that glutamatergic neurons are distributed mostly in the pars compacta (1, 32). The PPT besides glutamatergic neurons also contains numerous neurons including cholinergic and GABAergic neurons. It has been shown that these neurons are involved in cardiovascular regulation (12). For example, our previous study indicated that the GABA_A_ receptor and cholinergic neurons of this nucleus have an inhibitory effect on cardiovascular responses (17, 26). In addition, nitrergic neurons of the PPT have a hypotension effect (15). However, based on our results the glutamatergic neurons have an excitatory effect on blood pressure and an inhibitory effect on HR. At the present time these different effects of this nucleus on cardiovascular parameters are unknown. However, it has been reported that three types of neurons are present in this nucleus. Type 1 is characterized by continuous and fast-acting phase potentials that can be activated by the depolarization current and are probably glutamatergic (1, 33). Therefore, we suggest that glutamate increases the cardiovascular responses by activation of these neurons. It has been shown that PPT has two cholinergic and non-cholinergic projections to several brain areas including areas involved in cardiovascular regulation (12, 34, 35). It has been proposed that the non-cholinergic projections of PPT are glutamatergic or GABAergic. Because the PPT GABAergic neurons have short axons (36), we propose that the main projections are glutamatergic. Because a projection from the PPT to RVLM, an important sympatho‐excitatory area for cardiovascular regulation, has been reported (12, 14), we suggest that this projection is glutamatergic and plays an excitatory effect on cardiovascular responses (Figure 7). However, future studies are needed for this suggestion.

The role of the PPT nucleus in modulating arousal and defense responses has also been proposed. Defensive reactions are a natural reaction of the body to a threat, and threatening stimuli activate the sympathetic-adrenal system and inhibit the parasympathetic system. Therefore, we believe that the increase in blood pressure and tachycardia during stress is due to activation of the sympathetic system (37). A set of brain nuclei including PAG, amygdala (37), hypothalamic nuclei, and the CnF are involved in defense responses (38). Anatomical and immunohistochemically studies have shown that the PPT nucleus is associated with a large number of these regions, and its ascending outputs project to the superior regions of the brain and are involved in the integration of cardiovascular responses to specific conditions such as defense responses (37). These types of connections include the connection of the PPT nucleus with the PAG region and the hypothalamus, especially its lateral part (39). Due to the connections of the PPT nucleus with these areas, it is possible that during emotions and arousals, the activity of the glutamatergic system increases and causes an increase in blood pressure.

It has been reported that there is a local synaptic network in this nucleus in which the cholinergic and non-cholinergic neurons of this nucleus interfere with each other and create integrations between the inputs and outputs to the nucleus (14, 40). This localized neural network can be formed for any of the PPT activities. According to this theory, this local network may also exist in the case of cardiovascular activity. 

In addition, the presence of several important neurotransmitters such as GABA, acetylcholine, glutamate, and nitric oxide has been reported in the PPT (9). These neurotransmitters may be involved in integration of cardiovascular activity, although these probabilities are hypothetical and require further studies.

The relationship between pain and cardiovascular response such as blood pressure and tachycardia has been observed in previous studies (41). Many areas of the brain nucleus such as PAG, dorsal and medial hematopoietic nucleus, thalamus, locus coeruleus, paragigantocellular nucleus, and RVLM are involved in the integration of cardiovascular responses to the pain response (42). Previous studies have shown that the PPT nucleus responds to painful stimuli, especially painful stimulation of the animal’s tail, and increases the electrical activity of the nucleus (43). The PPT nucleus is also associated with the RVMM region, which is one of the most important areas of the descending pain modulation system. The nucleus also receives projections to the spinal cord (44). Because these layers of the spinal cord play a role in transmitting pain, it seems that pain inputs also enter this nucleus. The PPT nucleus also plays a role in controlling pain, since pain causes arousal along with decreased sleep, increased respiration rate, and increased blood pressure (45), it is suggested that neurons of the glutamatergic system of the PPT nucleus are activated during pain and cause increased blood pressure.

In this study MK-801 alone was microinjected into the PPT, it has been shown that this agent did not have a significant effect, therefore it is suggested in anesthetized condition release of glutamate is low or this type of receptor does not exist in this nucleus. To elucidate this suggestion, we co-injected MK801 + L-Glu. Results indicated that the cardiovascular effect of L-Glu was vigorously attenuated by MK-801, these results showed that the glutamatergic effect of the PPT on the cardiovascular system is mostly mediated by the NMDA receptor. This effect of L-Glu in the PPT is similar to previous studies. For example in the CnF nucleus, injection of AP5 (an antagonist of NMDA receptor) before L-Glu significantly attenuates the effect of L-Glu (23), which indicated the NMDA receptor is an important receptor involved in cardiovascular regulation.

It also reported that this nucleus responds to hypotension induced by hemorrhage. In a previous study, we showed that inactivation of the PPT with Cobalt chloride (COCl_2_) could attenuate tachycardia and hypotension induced by hemorrhage. This response of the PTT to hemorrhage allows us to suggest that its cardiovascular-related neurons are activated during hypotension. For further investigation the effect of the nucleus on hypotension was evaluated, Therefore, we induced hypotension with hydralazine, and the effect of the glutamatergic system on hypotension was investigated. The results showed that after induction of hypotension, microinjection of MK- 801 induced a depressor effect, and L-Glu injection also significantly ameliorated the hypotensive effect of HLZ. Based on this result we suggest that the glutamatergic system of the PPT nucleus plays a role in the regulation of cardiovascular parameters. However, we need additional research to investigate this effect of the glutamatergic system of the PPT. 

## Conclusion

Our findings indicated that activation of NMDA receptors of the glutamatergic system in the PPT elicits presser and bradycardia effects in both normotensive and hypotensive conditions. 

## Authors’ Contributions

MNS Contributed to the design and implementation of the research, analysis of the results, and writing of the manuscript. MRH Carried out the experiments. VH and HS were involved in planning and supervising the work. RM Helped supervise the project. AK Helped designe the study. All authors discussed the results and contributed to the final manuscript.

## Conflicts of Interest

The authors declare that there are no conflicts of interest.
